# Septic Bilateral Cavernous Sinus Thrombosis With Persistent Methicillin-Resistant Staphylococcus Aureus Bacteremia

**DOI:** 10.7759/cureus.8554

**Published:** 2020-06-10

**Authors:** Rajesh Kotagiri, Aaron T Saunders

**Affiliations:** 1 Internal Medicine, Banner University Medical Center, Tucson, USA

**Keywords:** septic cavernous sinus thrombosis, septic emboli, therapeutic anticoagulation, stroke, herniation, mechanical thrombectomy, heparin, trans-esophageal echocardiogram, methicillin-resistant staphylococcus aureus bacteremia

## Abstract

Septic cavernous sinus thrombosis (CST) is an extremely rare diagnosis that is characterized by nonspecific signs and symptoms. It is often precipitated by a recent facial or sinus infection, as the venous supply from these areas drains into the cavernous sinus. This case highlights significant morbidity and mortality in septic CST where all aggressive treatments did not lead to clinical improvement, and the precipitating cause of the thrombosis was never found. The patient reported herein decompensated despite several investigations and treatment measures due to the lack of proper evidence-based approach.

## Introduction

Septic cavernous sinus thrombosis (CST) is an extremely rare diagnosis that is characterized by nonspecific signs and symptoms. It is often precipitated by a recent facial or sinus infection [1-3], as the venous supply from these areas drains into the cavernous sinus. As exemplified in the case presented herein, patients with septic CST can present atypically without any known precipitating infection and can be refractory to many aggressive treatments such as anticoagulation and antibiotic therapy, leading to poor patient outcomes.

## Case presentation

**Figure 1:**
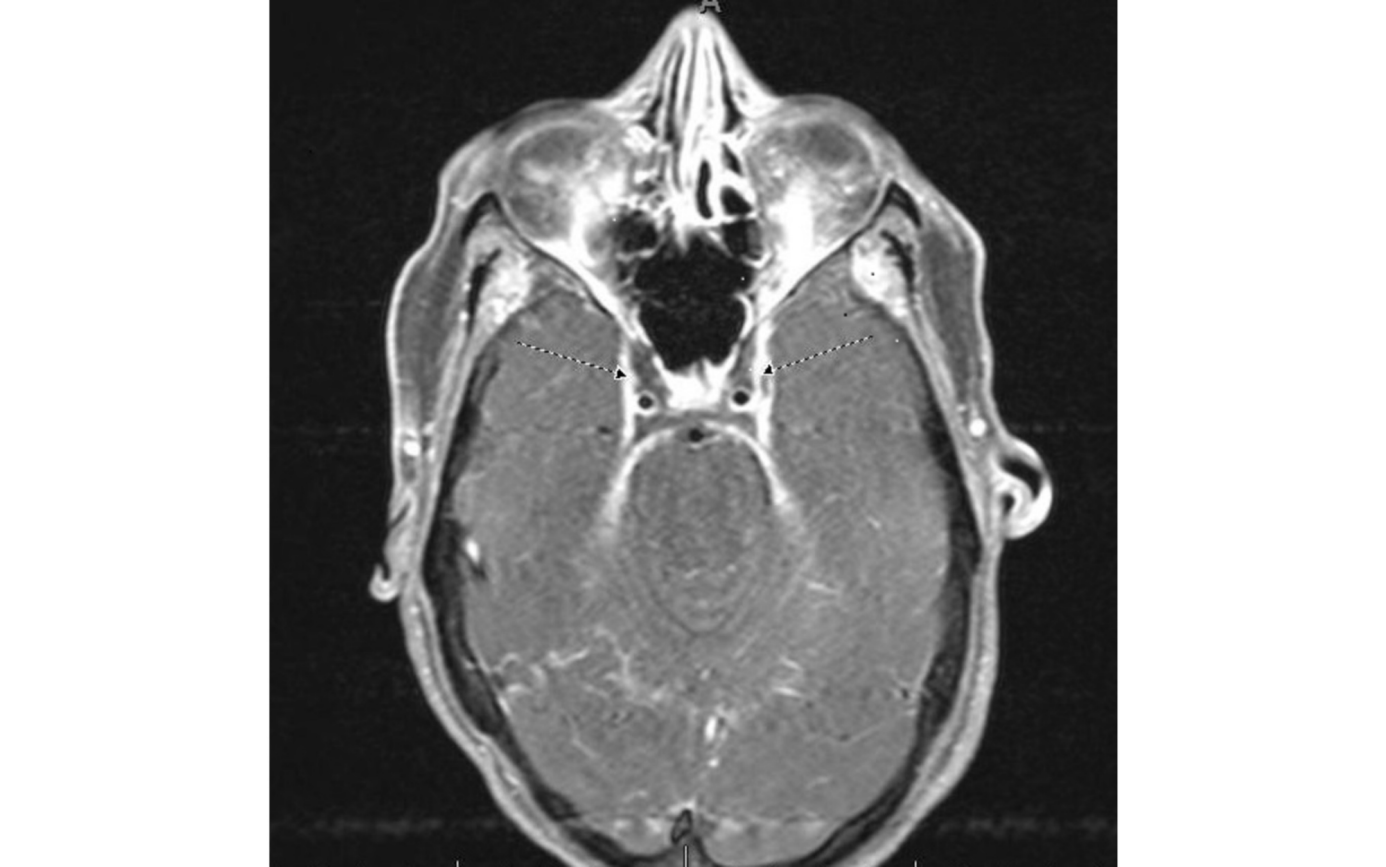
MRI of the brain/orbits, with and without contrast, showing bilateral cavernous sinus thrombosis (dotted arrows) with left retrobulbar enhancement and no evidence of sinusitis.

A 35-year-old female with a history of methamphetamine abuse presented with a three- to four-day history of neck stiffness associated with sharp neck pain, subjective fevers, headaches, diplopia, and left-sided ptosis. Initial vitals were normal, and the physical examination was remarkable for right-sided cranial nerve (CN) VI (lateral gaze) palsy and left partial CN III palsy. The head CT scan was normal. Out of concern for meningitis, a lumbar puncture was performed. Cerebrospinal fluid (CSF) analysis was unremarkable. There were no concerns for auto-immune diseases. Vancomycin with a trough goal of 15-20 mcg/mL and ceftriaxone 2 gm every 12 hours were empirically started for central nervous system (CNS) infection, despite the normal CSF analysis. MRI of the brain/orbits, with and without contrast, revealed bilateral CST with left retrobulbar enhancement, with no evidence of sinusitis (Figure 1).

**Figure 2:**
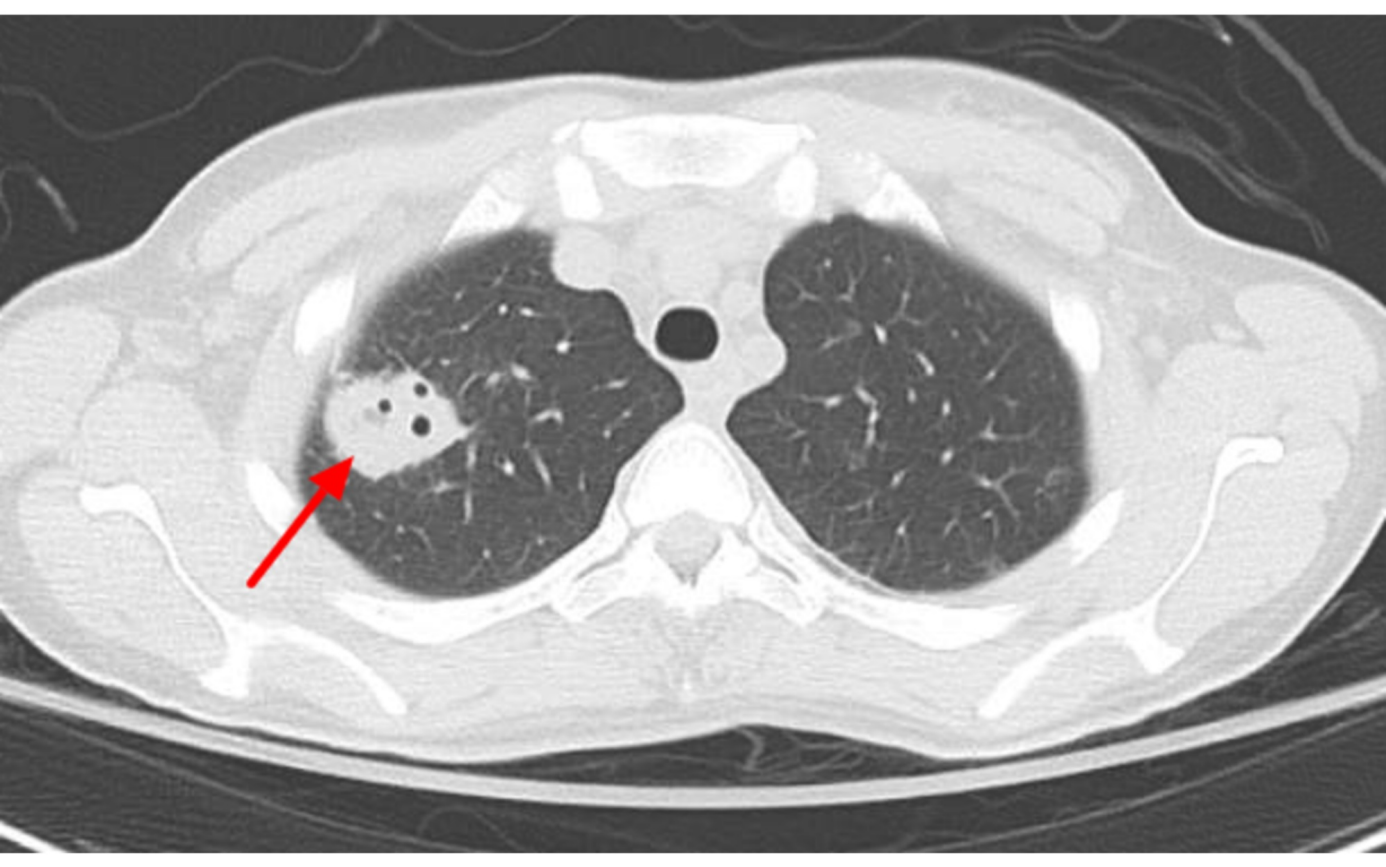
Non-contrast chest CT showed cavitary lesions (red arrow) on the right lung (largest ~3 cm) suggestive of septic emboli.

**Figure 3:**
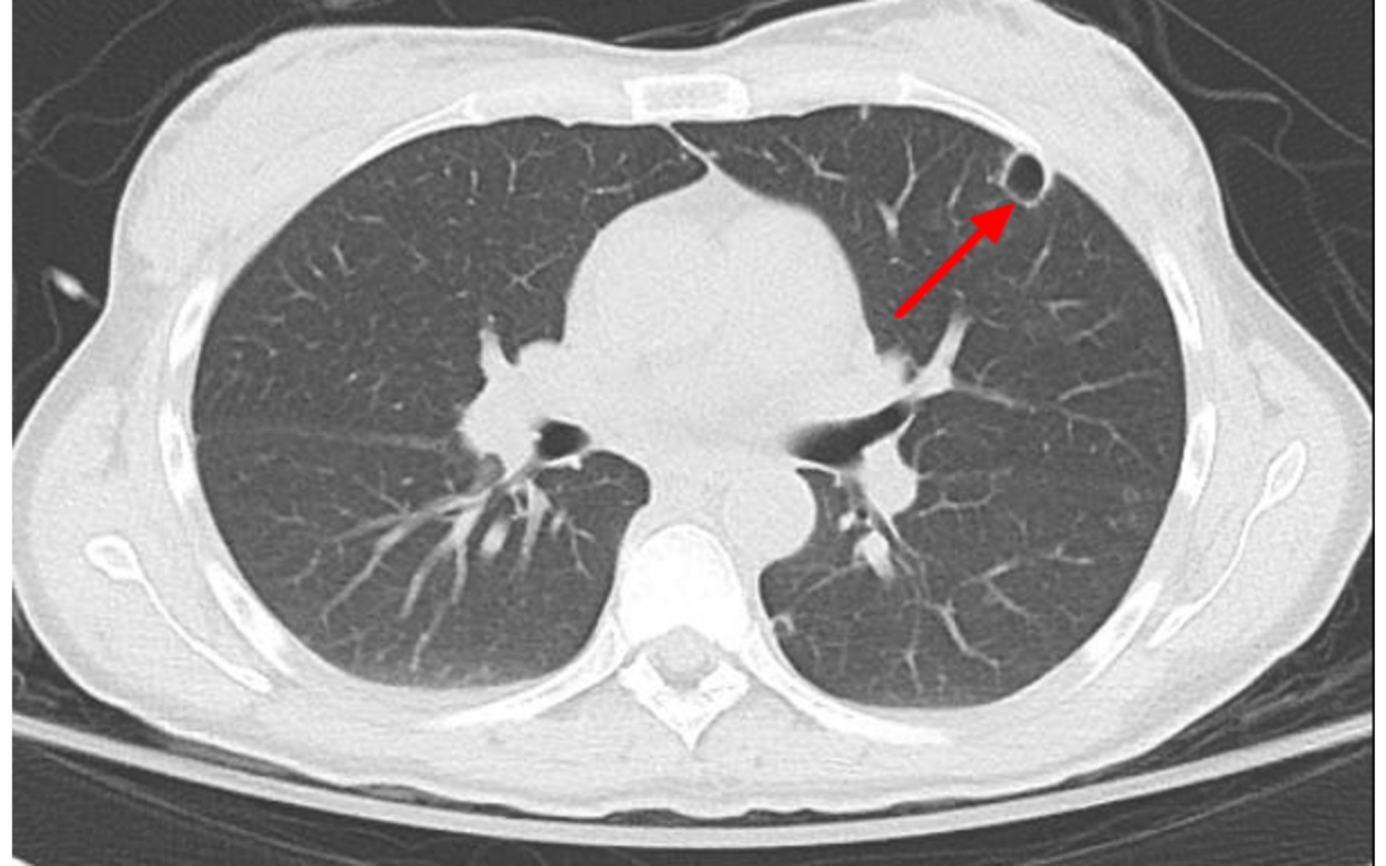
Non-contrast CT chest showing a left cavitary lesion (red arrow) on the left lung.

Heparin infusion was started. Initial blood cultures eventually returned positive for Methicillin-resistant Staphylococcus aureus (MRSA). Vancomycin was continued and ceftriaxone was stopped. Both transthoracic echocardiogram (TTE) and transesophageal echocardiogram (TEE) did not reveal valvular vegetations or masses. MRI of the entire spine was obtained and was negative for the presence of an abscess, osteomyelitis, and discitis. Over several days, the patient became progressively more encephalopathic and febrile with fevers greater than 39 degrees Celsius. Serial blood cultures revealed persistent MRSA bacteremia for about one week, at which point ceftaroline was started for dual antibiotic coverage for MRSA. Vancomycin was eventually switched for linezolid with the goal of improving CNS penetration. The patient developed a drug rash to ceftaroline and was therefore switched to daptomycin. Chest CT without contrast showed findings suggestive of multiple septic emboli throughout both the lungs (Figures 2, 3).

**Figure 4:**
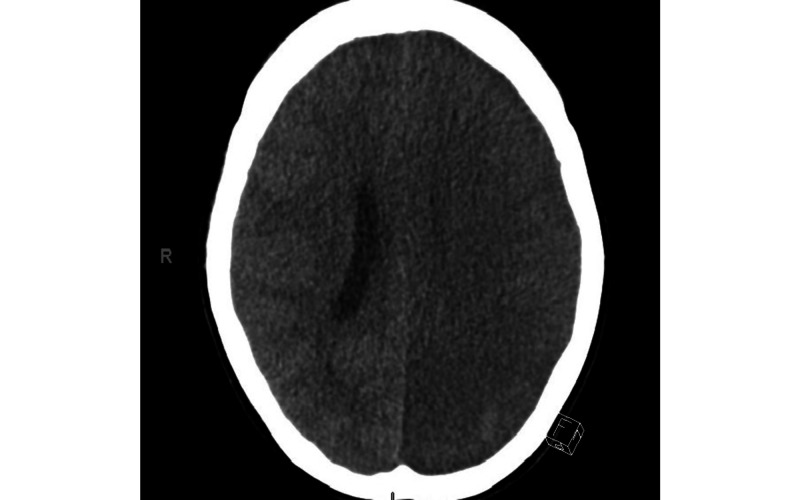
CT scan of the brain showing diffuse cerebral edema with effacement of the basal cisterns and 5-mm rightward midline shift with concerns for impending herniation.

The blood cultures were negative for a few days. About two weeks into her hospitalization, the patient developed sudden onset of right-sided weakness. Head CT showed two hypodense infarcts in the left occipital lobe and thalamus/basal ganglia, consistent with left acute non-hemorrhagic posterior cerebral artery territory infarct, the findings of which were supported by MRI. Thrombolysis was contraindicated as the patient was already therapeutically anticoagulated. The patient became progressively more septic and encephalopathic and was intubated. Repeat head CT revealed interval progression and a small amount of hemorrhagic conversion of the infarcts, expansion of the bilateral CST, and diffuse cerebral edema with effacement of the basal cisterns and 5-mm rightward midline shift, concerning for herniation (Figure 4).

The subsequent examination was consistent with brain death, and life support was discontinued.

## Discussion

This case exemplified the quick progression of a rare diagnosis, and, in retrospect, many questions remain unanswered. There was no evidence of precipitating facial or sinus infection in this case, whereas most other cases noted in the literature are associated with a precipitating infection. No definitive source of MRSA bacteremia was ever found; however, inoculation by IV drug use was a possibility given the history of substance abuse. MRSA bacteremia persisted for over a week despite dual antibiotic coverage, possibly due to the thrombosis serving as a nidus for infection. The thrombosis continued to expand despite therapeutic anticoagulation. The patient had evidence of multiple septic emboli to the lungs and developed left posterior acute non-hemorrhagic stroke (likely embolic) despite both TTE and TEE being negative for any evidence of endocarditis. Overall, there are only a few retrospective small studies on the management of CST due to the rarity of this syndrome. Anticoagulation (unfractionated heparin or low-molecular-weight heparin) has only been shown to decrease morbidity, ophthalmoplegia, blindness, stroke, and seizures, but it has not been shown to have overall mortality benefit [4]. Mortality for this condition is high and estimated around 30% based on limited studies [1]. Optimal duration of treatment with anticoagulation has not been established. In severe cases, limited evidence has shown no significant benefit to endovascular thrombolysis or mechanical thrombectomy compared with the treatment with anticoagulation [5,6].

## Conclusions

In summary, this case highlights significant morbidity and mortality in septic CST, an extremely rare diagnosis in clinical practice. It also uniquely demonstrates a case where all aggressive treatments did not lead to clinical improvement, and the precipitating cause of the thrombosis was never found.
